# Convenient Synthesis and Antimicrobial Activity of Some Novel Amino Acid Coupled Triazoles

**DOI:** 10.3390/molecules15106759

**Published:** 2010-09-28

**Authors:** Samir Mohamed El Rayes

**Affiliations:** Department of Chemistry, Faculty of Science, Suez Canal University, Ismailia, Egypt; E-Mail: samir_elrayes@yahoo.com; Tel.: +2127500914; Fax: +2643202381

**Keywords:** triazoles, azide coupling, peptides, Curtius

## Abstract

This study describes a promising one-pot synthesis of [2-(5-benzyl-4-phenyl-4H-[1,2,4]triazol-3-thio)-acetyl]-amino acid methyl esters **6a-h** and dipeptides **10a-e,** which were successfully synthesized starting from amino acid esters **5a-h**, **9a-e** and azides **4**, **8a,b**, respectively. On the other hand, azide **4** underwent Curtius rearrangement to the corresponding isocyanate, which subsequently reacted with selected aliphatic amine and/or aniline derivatives to give the corresponding urea derivatives **11** and **12a,b**. Reactions of the isocyanate with secondary amines gave amide derivatives **13a,b**. The structural elucidation of products is reported and some of the products were also screened for their antimicrobial activity.

## 1. Introduction

The emergence of drug resistance in disease treatment calls for the continuing availability of new chemotherapeutic agents able to overcome this problem. In the last few decades, the chemistry of 1,2,4-triazoles and their fused heterocyclic derivatives have received considerable attention due to their synthetic and effective biological importance. Based on the principle of additive activity, we anticipated obtaining reinforcement of biological activities by means of substitutions at different positions of amino acid substituted-1,2,4-triazole derivatives. On the other hand; the electronic structure of sulfur imbues sulfurous organic compounds, including amino acids, with chemical reactivities beyond those of the corresponding oxygen or nitrogen-containing analogs. Biosulfur compounds are much more nucleophilic and acidic than their oxygen analogs, allowing thiols (RSH) and thioethers (R-S-R') to participate in a far greater range of electrophilic substitutions than the corresponding alcohols [1]. 

**Figure 1:**
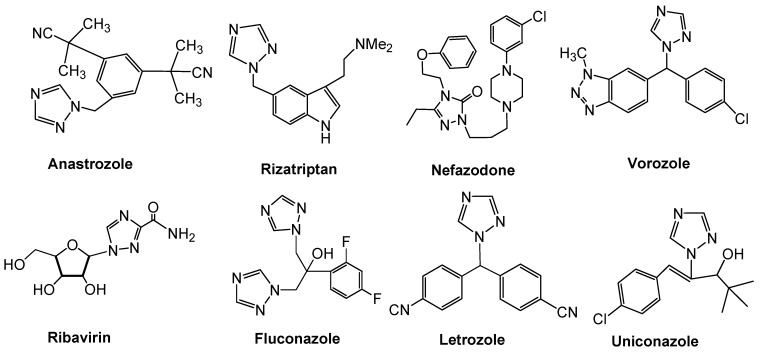
Biologically active triazoles.

Recently the literature has reported a large number of 1,2,4-triazole-containing ring systems that have been incorporated into a wide variety of therapeutically interesting drug candidates including antiseptic, analgesic, anti-convulsant [2,3,4,5,6,7,8,9,10,11,12,13], antibiotic [2], antiallergic [2], anti-inflammatory [2,3,4,5,6,7,8,9,10,11,14], diuretic [2,6,9], fungicidal [4,5,11,12,13,14], insecticidal [4,11,14], herbicidal [4,11,14], antibacterial [4,5,6,7,12,13], antiviral [3,4,5,6,8,9,11], antidepressant [3,6,10], antimicrobial [3,4,5,6,8,11,12,13], antitumor [4,7,10,11], antihypertensive [6,9,10], and antimigraine compounds [8]. In addition there are several well known drugs, e.g. anastrozole, rizatriptan, nefazodone, vorozole, ribavirin, fluconazole, letrozole and uniconazole, that contain the 1,2,4-triazole group (Figure 1). 

On the other hand, many triazole derivatives also have industrial applications as precursors for photosensitive materials (*i.e.*, inks and toners) [15], in polymer chemistry [12], and others [16,17]. Unfortunately, the water solubility of most triazole compounds is too poor for use in a clinical trials as medicines [18]. In this paper, we describe the development of a new series of 1,2,4-triazole derivatives, whose chemical modifications include coupled amino acid and dipeptide derivatives. 

## 2. Results and Discussion

### 2.1. Chemistry

The synthesis of new amino acid derivatives coupled with biologically active heterocyclic moieties such as triazoloquinazoline [19], quinoline [20], and pyradizinone [21] attracted our attention. In this work we studied 5-benzyl-4-phenyl-2,4-dihydro-3H-1,2,4-triazole-3-thione (**1**) as a biologically active heterocyclic precursor which was synthesized according to the established procedure [3].

The hydrazide **3** could be prepared from **1** by regioselective *S*-alkylation [22] with ethyl chloroactetate to give the corresponding ester **2**, which was subsequently hydrazinolyzed by hydrazine hydrate. 

**Scheme 1:**
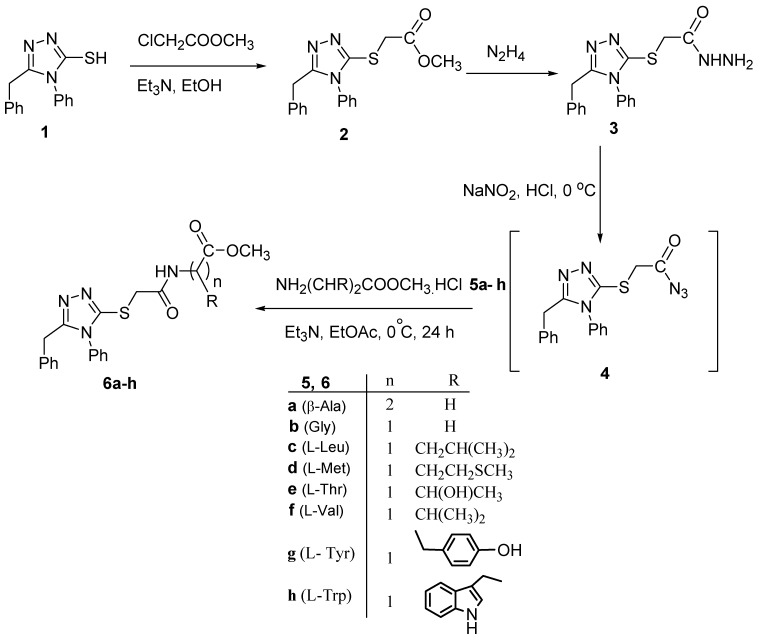
Synthesis of amino acid monopeptides coupled with triazole.

The acyl azide pathway was one of the first peptide coupling methods developed by Curtius [23]. Synthesis of the target amino acid derivatives **6a-h** were successfully achieved *via* the azide coupling method [19,20,21,24], which was reported to minimize the degree of racemization in amino acid coupling. An ethyl acetate solution of the *in situ* generated azide **4** reacted with the amino acid methyl ester hydrochlorides **5a-h** in the presence of triethylamine to afford [2-(5-benzyl-4-phenyl-4*H*-[1,2,4]triazol-3-thio)-acetyl]-amino acid methyl esters **6a-h** in good to moderate yields (Scheme 1). 

**Scheme 2:**
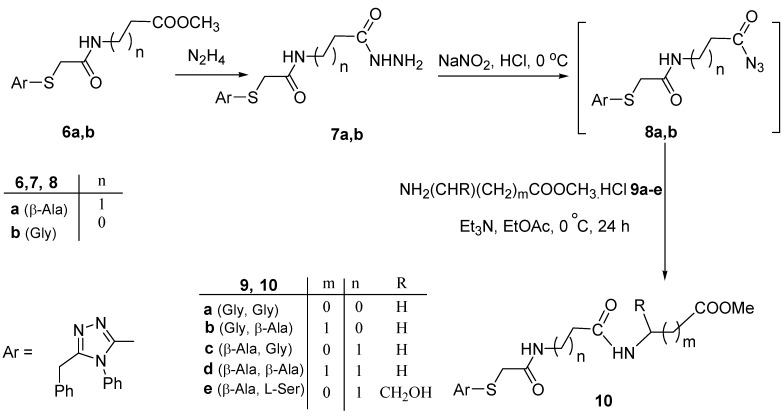
Synthesis of amino acid dipeptides coupled with triazole.

Further development of the azide coupling was achieved by the synthesis of *N*-substituted dipeptide derivatives **10a-e**. Thus, boiling the amino acid ester derivatives **6a,b** (β-Ala, Gly) with hydrazine hydrate gave the acyl hydrazides **7a, b** (Scheme 2). Finally, nitrosation of acyl hydrazides **7a, b** by treatment with a NaNO_2_ and HCl mixture gave the acyl azides **8a,b**. The *in situ* generated azides **8a, b** reacted with amino acid methyl ester hydrochlorides **9a-e** in ethyl acetate in the presence of triethylamine to produce dipeptide derivatives **10a-e** in reasonable yield (Scheme 2). 

**Scheme 3:**
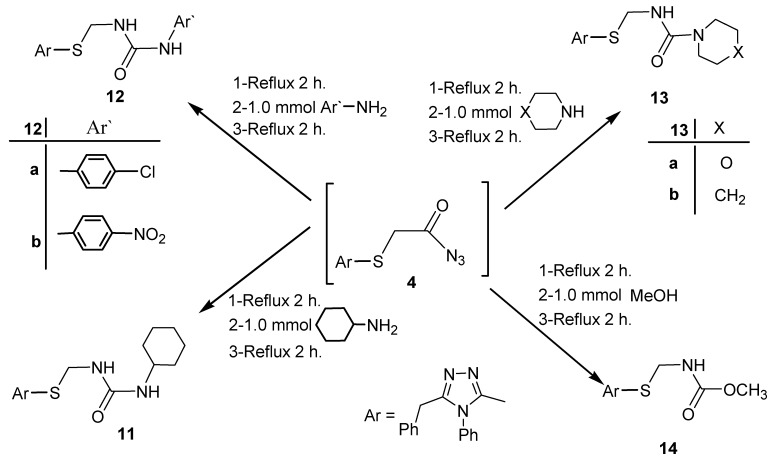
Synthesis of triazole derivatives under Curtius rearrangement condition.

An extension of this study was achieved by refluxing the azide **4** in a non polar solvent such as benzene whereupon a Curtius rearrangement occurred to give the corresponding isocyanate. On treatment *in situ* of the isocyanate with selected aliphatic amine and/or aniline derivatives urea derivatives **11** and **12a,b** were obtained. The reaction of isocyanate with secondary amines gave amide derivatives **13a,b**, whereas with methanol it gave the carbamic acid derivative **14** (Scheme 3). 

**Figure 2:**
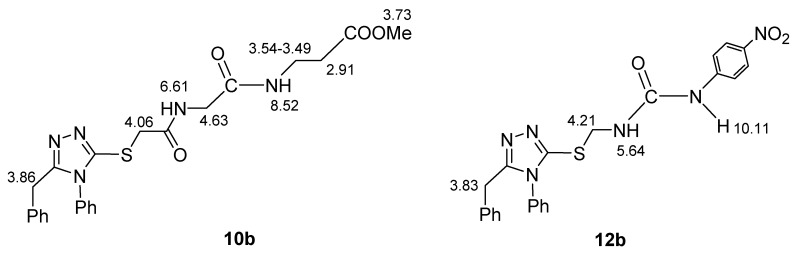
Selected ^1^H-NMR assignments of compounds **10b** and **12b**.

The structural assignment of ester **2**, acyl hydrazide **3,** *N*-substituted amino acid esters **6a-h**; acyl hydrazides **7a,b**, *N*-substituted dipeptides **10a-e**, urea derivatives **11** and **12a,b**, amide derivatives **13a,b** and carbamic acid derivative **14** is based on ^1^H-NMR,^13^C-NMR, IR, mass spectral and physicochemical analyses. The ^1^H-NMR spectrum of the *N*-substituted dipeptide **10b**, for example, exhibits signals at *δ* 8.52, 6.61, 4.63, 3.73, 3.54-3.49 and 2.31 ppm corresponding to the functionalities found at the dipeptide chain; two NH groups, CH_2_ (glycyl residue), OMe of ester and two CH_2_ (β-alanine residue), respectively (Figure 2). The ^1^H-NMR spectrum of the urea derivative **12b** showed two characteristic signals at *δ* 10.11 and 5.64 ppm for the two NH groups. The ^1^H-NMR spectra of all compounds showed two characteristic signals, one within the range *δ* 4.37-4.00 ppm for SCH_2_ and the other within *δ* 412- 3.80 ppm for PhCH_2_ (Figure 2). 

### 2.2. Antimicrobial studies

The antimicrobial activity of sixteen triazole derivatives was assayed by the agar well diffusion method [25] against two bacterial colonies (*Escherechia coli* and *Bacillus subtilis*) and two fungal cultures (*Phytophthora infestans* and *Colletotricum gloeosporioides*). Five-millimeter diameter wells were cut out in agar plates using a sterile cork-borer. Fifty µL of 4 mg/mL test solutions were transferred aseptically to the wells. Plates were incubated at 25 °C for 24 hours and four days for bacteria and fungi, respectively. The antimicrobial activity was evaluated by measuring the inhibition zone formed around the wells. Wells containing sterile distilled water or the solvent (ethanol) served as controls. Results are listed in Table 1.

The results showed that not all derivatives were active against the tested microorganisms. Derivative **2** inhibited the growth of *P. infestans*, but it failed against *C. gloeosporioides* and the two bacterial organisms (*E. coli*, *B. subtilis*.). On the other hand, **12b** was effective against *C. gloeosporioides*, but not against *P. infestans* (Table 1). As for bacteria, derivatives **6c** and **12b** inhibited the growth of the bacterium *B. subtilis*, while derivative No. **14** was inhibitory to the growth of the bacterium *E. coli* (Table 1). It is worth mention that derivative **12b** was effective against both types of microorganisms (*B. subtilis* and *C. gloeosporioides*) (Table 1).

## 3. Experimental

### 3.1. General

Solvents were purified and dried in the usual way. The boiling range of the petroleum ether used was 40–60 °C. Thin layer chromatography (TLC): silica gel 60 F_254_ plastic plates (E. Merck, layer thickness 0.2 mm) detected by UV absorption. Elemental analyses were performed on a *Flash EA-1112* instrument at the Microanalytical Laboratory, Faculty of Science, Suez Canal University, Ismailia, Egypt. Melting points were determined on a Buchi 510 melting-point apparatus and the values are uncorrected. IR spectra measured with Perkin Elmer 1430 ratio recording. NMR spectra were measured with Bruker spectrometers (200 MHz and 300 MHz) and TMS (0.00 ppm) was used as internal standard. The mass spectra were measured with a KRATOS Analytical Kompact spectrometer. The starting compound **1** was prepared according to a described method [3].

### 3.2. (5-Benzyl-4-phenyl-4H-[1,2,4]triazol-3-thio)-acetic acid ethyl ester (***2***)

A solution of 5-benzyl-4-phenyl-2,4-dihydro-3*H*-1,2,4-triazole-3-thione (**1,** 0.27 g, 1.0 mmol) in ethanol (30 mL), Et_3_N (0.20 mL, 2.0 mmol) and chloroacetic acid ethyl ester (0.12 mL, 1.0 mmol) was mixed and then refluxed for 24 h while the reaction was monitored via tlc. The reaction mixture was then cooled and an ice/water mixture was added. The syrup formed was extracted with cold ethyl acetate (30 mL); washed with 2 N Na_2_CO_3_ and dried (Na_2_SO_4_). The solvent was evaporated under vacuum to obtain a pure oily product. Colorless oil (0.31 g, 88%); IR (neat): 3063, 2971, 1732, 1673, 1661, 1620, 1510, 1460, 1403 cm^-1^; ^1^H-NMR (200 MHz, CDCl_3_ *δ* ppm): 7.61-7.52 (4H, m, ArH); 7.29-7.17 (4H, m, ArH); 7.07 (2H, d, *J* = 8.4 Hz, ArH); 4.31 (2H, s, SCH_2_); 4.12 (2H, s, PhCH_2_); 3.97 (2H, q, *J* = 7.2 Hz, OCH_2_CH_3_); 1.36 (3H, t, *J* = 7.2 Hz, OCH_2_CH_3_). Anal. Calcd. For C_19_H_19_N_3_O_2_S (353.12): C, 64.57; H, 5.42; N, 11.89; S, 9.07; Found: C, 64.36; H, 5.38; N, 11.78; S, 8.99.

### 3.3. (5-Benzyl-4-phenyl-4H-[1,2,4]triazol-3-thio)-acetic acid hydrazide (***3***)

To a solution of **2** (0.35 g, 1.0 mmol) in ethanol (30 mL), hydrazine hydrate (0.24 mL, 5.0 mmol) was added. The reaction mixture was refluxed for 4h, afterwards it was stirred overnight at room temperature; cold water was added, the formed precipitate was filtered off, washed with ethanol and ether then crystallized from aqueous ethanol to yield the hydrazide **3**. Colorless crystals (0.32 g, 94%); mp 167–168 °C, IR (KBr disk): 3310, 3290, 3205, 3074, 2961, 1676, 1668, 1652, 1604, 1540, 1502, 1451, 1400 cm^-1^; ^1^H-NMR (200 MHz, CDCl_3_ *δ* ppm): 8.42 (1H, bs, D_2_O exchangeable, NH); 7.63-7.57 (4H, m, ArH); 7.31-7.22 (4H, m, ArH); 7.13 (2H, d, *J* = 8.2 Hz, ArH); 4.37 (2H, s, SCH_2_); 4.33 (2H, bs, D_2_O exchangeable, NH_2_); 4.08 (2H, s, PhCH_2_). Anal. Calcd. For C_17_H_17_N_5_OS (339.12): C, 60.16; H, 5.05; N, 20.63; S, 9.45; Found: C, 60.32; H, 5.19; N, 20.48; S, 9.65.

### 3.4. General procedure for azide method; preparation of ***6a-h***

To a cold solution (−5 °C) of hydrazide **3** (0.34 g, 1.0 mmol) in acetic acid (6 mL), 1 N HCl (3 mL), and water (25 mL) was added a solution of NaNO_2_ (0.87 g, 1.0 mmol) in cold water (3 mL). The reaction mixture was stirred at −5 °C for 15 min. The yellow syrup formed was extracted with cold ethyl acetate (30 mL), washed with cold 3% NaHCO_3_, H_2_O and finally dried (Na_2_SO_4_). To this solution amino acid esters **5a-h** (1.0 mmol) in ethyl acetate (20 mL) containing 0.2 mL of triethylamine was added. The reaction mixture was kept at −5 °C for 24 h., then at 25 °C for another 24 h. The solution was evaporated to dryness, and the residue was crystallized from petroleum ether/ ethyl acetate to give the desired product.

*Methyl-3-[2-(5-benzyl-4-phenyl-4H-[1,2,4]triazol-3-thio)-acetylamino]-propionate* (**6a)**. From β-AlaOCH_3_·HCl (**5a**, 0.14 g). Colorless crystals (0.29 g, 71 %); mp 102 °C, IR (KBr disk): 3271, 3061, 2969, 1757, 1695, 1680, 1663, 1607, 1536, 1506, cm^-1^; ^1^H-NMR (200 MHz, CDCl_3_ *δ* ppm): 8.16 (1H, bs, D_2_O exchangeable, NH); 7.54-7.35 (4H, m, ArH); 7.28-7.17 (4H, m, ArH); 7.08 (2H, d, *J* = 8.2 Hz, ArH), 4.09 (2H, s, SCH_2_); 3.88 (2H, s, PhCH_2_); 3.74 (3H, s, OMe); 3.64-3.59 (2H, m, CH_2_NH); 2.63 (2H, t, *J* = 7.2 Hz, CH_2_CO_2_Me), ^13^C-NMR (CDCl_3_, 75 MHz, *δ* ppm): *δ* 32.3, 33.7, 35.5, 38.4, 51.2, 127.9, 128.1, 128.4, 128.8, 129.0, 129.6, 130.5, 137.8, 144.0, 159.0, 171.4, 173.7; Anal. Calcd. For C_21_H_22_N_4_O_3_S (410.14): C, 61.44; H, 5.40; N, 13.65; S, 7.81; Found: C, 61.28; H, 5.53; N, 13.87; S, 7.96. Mass spectrum, m/z (%): 411(3), 410(11), 333(19), 319(28), 281(14), 280(43), 266(19), 267(53), 235(38), 234(26), 91(100).

*Methyl-2-[2-(5-benzyl-4-phenyl-4H-[1,2,4]triazol-3-thio)-acetylamino]-acetate* (**6b**). From GlyOCH_3_·HCl (**5b**, 0.13 g). Colorless crystals (0.27 g, 68 %); mp 114 °C, IR (KBr disk): 3298, 3081, 2973, 1767, 1695, 1672, 1649, 1622, 1541, 1492, cm^-1^; ^1^H-NMR (200 MHz, CDCl_3_ *δ* ppm): 8.51 (1H, bs, D_2_O exchangeable, NH); 7.55-7.52 (4H, m, ArH); 7.35-7.24 (4H, m, ArH); 7.11 (2H, d, *J* = 8.2 Hz, ArH); 4.11 (2H, s, SCH_2_); 4.01 (2H, d, *J* = 7.2 Hz, CH_2_CO_2_Me); 3.98 (2H, s, PhCH_2_); 3.81 (3H, s, OMe). ^13^C-NMR (CDCl_3_, 75 MHz, *δ* ppm): *δ* 29.7, 37.8, 44.1, 50.6, 127.6, 128.2, 128.5, 128.8, 129.3, 129.9, 130.3, 137.9, 145.3, 158.6, 171.0, 173.4; Anal. Calcd. For C_20_H_20_N_4_O_3_S (396.13): C, 60.59; H, 5.08; N, 14.13; S, 8.09; Found: C, 60.48; H, 5.22; N, 14.07; S, 8.18. Mass spectrum, m/z (%): 397(5), 396(9), 319(16), 305(23), 281(13), 280(48), 266(23), 267(61), 234(19), 235(49), 91(100).

*Methyl-2-[2-(5-benzyl-4-phenyl-4H-[1,2,4]triazol-3-thio)-acetylamino]-4-methyl-pentanoate* (**6c**). From l-LeuOCH_3_·HCl (**5c**, 0.18 g). Colorless crystals (0.34 g, 75 %); mp 94 °C, IR (KBr disk): 3256, 3043, 2955, 1751, 1700, 1681, 1658, 1600, 1561, 1520, 1500 cm^-1^; ^1^H NMR (200 MHz, CDCl_3_ *δ* ppm): 8.33 (1H, bs, D_2_O exchangeable, NH); 7.54-7.38 (4H, m, ArH); 7.32-7.23 (2H, m, ArH); 7.10-7.04 (4H, m, ArH); 4.65-4.62 (1H, m, NHCH); 4.11 (2H, s, SCH_2_); 3.97 (2H, s, PhCH_2_); 3.79 (3H, s, OMe); 1.76-1.72 (2H, m, NHCH_2_); 1.36-1.34 (1H, m, CH_2_CH); 1.01 (6H, d, *J* = 6.8 Hz, (CH_3_)_2_CH), ^13^C-NMR (CDCl_3_, 75 MHz, *δ* ppm) 20.4, 21.6, 32.4, 38.5, 39.3, 48.9, 50.4, 128.0, 128.1, 128.3, 128.5, 129.0, 129.9, 130.0, 137.7, 144.2, 158.8, 172.5, 174.6. Anal. Calcd. For C_24_H_28_N_4_O_3_S (452.19): C, 63.69; H, 6.24; N, 12.38; S, 7.09; Found: C, 63.52; H, 6.11; N, 12.21; S, 6.85. Mass spectrum, m/z (%): 453(2), 452(6), 375(13), 361(18), 281(19), 280(52), 266(25), 267(71), 234(17), 235(33), 91(100).

*Methyl-2-[2-(5-benzyl-4-phenyl-4H-[1,2,4]triazol-3-thio)-acetylamino]-4-(methylthio)-butyrate* (**6d**)**.** From l-MetOCH_3_·HCl (**5d**, 0.20 g). Colorless crystals (0.29 g, 62%); mp 89 °C, IR (KBr disk): 3262, 3061, 2973, 1764, 1689, 1677, 1649, 1605, 1554, 1511, 1485cm^-1^; ^1^H-NMR (200 MHz, CDCl_3_ *δ* ppm): 8.51 (1H, bs, D_2_O exchangeable, NH); 7.55-7.51 (4H, m, ArH); 7.37-7.26 (2H, m, ArH); 7.12-7.04 (4H, m, ArH); 4.76-4.73 (1H, m, NHCH); 4.11 (2H, s, SCH_2_); 3.96 (2H, s, PhCH_2_); 3.81 (3H, s, OMe); 2.59 (2H, t, *J* = 7.2 Hz, CH_2_SMe); 2.26-2.24 (2H, m, CHCH_2_); 2.15 (3H, s, SMe). ^13^C-NMR (CDCl_3_, 75 MHz, *δ* ppm): *δ* 17.2, 27.9, 29.6, 32.7, 38.0, 51.2, 54.0, 127.6, 128.5, 128.7, 128.8, 129.6, 129.8, 131.0, 137.9, 145.2, 159.6, 171.7, 173.1; Anal. Calcd. For C_23_H_26_N_4_O_3_S_2_ (470.61): C, 58.70; H, 5.57; N, 11.91; S, 13.63; Found: C, 58.58; H, 5.43; N, 11.78; S, 13.79. Mass spectrum, m/z (%): 471(3), 470(8), 423(16), 409(17), 396(28), 319(19), 305(27), 281(22), 280(56), 266(32), 267(64), 234(23), 235(26), 91(100).

*Methyl-2-[2-(5-benzyl-4-phenyl-4H-[1,2,4]triazol-3-thio)-acetylamino]-3-hydroxy-butyrate* (**6e**)**.** From l-ThrOCH_3_·HCl (**5^e^**, 0.17 g). Colorless crystals (0.23 g, 52%); mp 101 °C, IR (KBr disk): 3522, 3291, 3055, 2967, 1746, 1704, 1680, 1649, 1610, 1544, 1507 cm^-1^; ^1^H-NMR (200 MHz, CDCl_3_ *δ* ppm): 8.28 (1H, bs, D_2_O exchangeable, NH); 7.57-7.53 (4H, m, ArH); 7.38-7.28 (2H, m, ArH); 7.13-7.03 (4H, m, ArH); 4.67-4.63 (1H, m, NHCH); 4.44 (1H, bs, D_2_O exchangeable, HCOH); 4.32-4.28 (1H, m, CH_3_CH); 4.12 (2H, s, SCH_2_); 4.00 (2H, s, PhCH_2_); 3.85 (3H, s, OMe); 1.37 (3H, d, *J* = 6.8 Hz, Me). ^13^C-NMR (CDCl_3_, 75 MHz, *δ* ppm): *δ* 17.9, 27.6, 38.7, 51.6, 60.9, 66.9. 127.2, 128.3, 128.4, 128.6, 129.2, 129.9, 132.2, 138.0, 145.7, 158.9, 170.9, 172.8; Anal. Calcd. For C_22_H_24_N_4_O_4_S (440.52): C, 59.98; H, 5.49; N, 12.72; S, 7.28; Found: C, 60.27; H, 5.63; N, 12.56; S, 7.09. Mass spectrum, m/z (%): 441(5), 440(10), 422(19), 396(34), 319(21), 305(32), 281(25), 280(47), 266(29), 267(71), 234(19), 235(22), 91(100).

*Methyl-2-[2-(5-benzyl-4-phenyl-4H-[1,2,4]triazol-3-thio)-acetylamino]-3-methyl-butyrate* (**6f**)**.** From l-ValOCH_3_·HCl (**5f**, 0.12 g). Colorless crystals (0.33 g, 75%); mp 93 °C, IR (KBr disk): 3283, 3072, 2981, 1761, 1707, 1680, 1649, 1610, 1572, 1545, 1523 cm^-1^; ^1^H-NMR (200 MHz, CDCl_3_ *δ* ppm): 8.25 (1H, bs, D_2_O exchangeable, NH); 7.46-7.38 (4H, m, ArH); 7.22-7.16 (2H, m, ArH); 6.97-6.91 (4H, m, ArH); 4.45-4.41 (1H, m, NHCH); 4.00 (2H, s, SCH_2_); 3.86 (2H, s, PhCH_2_); 3.68 (3H, s, OMe); 2.19 (1H, m, CH_3_CH); 0.92-0.90 (6H, d, *J* = 6.8 Hz, CH(CH_3_)_2_). ^13^C-NMR (CDCl_3_, 75 MHz, *δ* ppm): *δ* 17.0, 26.9, 28.3, 39.7, 51.0, 62.1, 127.0, 128.5, 128.6, 128.8, 129.5, 130.2, 131.8, 138.2, 145.3, 159.6, 170.4, 172.8; Anal. Calcd. For C_23_H_26_N_4_O_3_S (438.54): C, 62.99; H, 5.98; N, 12.78; S, 7.31; Found: C, 63.11; H, 6.13; N, 12.59; S, 7.26.

*Methyl-2-[2-(5-benzyl-4-phenyl-4H-[1,2,4]triazol-3-thio)acetylamino]-3-(4-hydroxyphenyl)-propionate* (**6g**). From l-TyrOCH_3_·HCl (**5g**, 0.18 g). Colorless crystals (0.32 g, 64%); mp 117 °C, IR (KBr disk): 3555, 3281, 3092, 2972, 1738, 1697, 1684, 1667, 1613, 1563, 1507 cm^-1^; ^1^H-NMR (200 MHz, CDCl_3_ *δ* ppm): 8.40 (1H, bs, D_2_O exchangeable, NH); 7.78-7.75 (4H, m, ArH); 7.61-7.49 (4H, m, ArH); 7.27-7.08 (6H, m, ArH); 5.76-5.59 (1H, bs, D_2_O exchangeable, OH); 5.12-4.97 (1H, m, NHCHCH_2_); 4.37 (2H, s, SCH_2_); 4.14 (2H, s, PhCH_2_); 4.03 (3H, s, OMe); 3.41-3.32 (2H, d, *J* = 6.8 Hz, CH_2_CH), ^13^C-NMR (CDCl_3_, 75 MHz, *δ* ppm) 31.8, 36.6, 37.8, 51.7, 56.4, 121.3, 127.7, 127.9, 128.5, 128.8, 128.9, 129.0, 129.2, 129.4, 132.8, 136.1, 145.4, 157.3, 160.1, 171.3, 173.4; Anal. Calcd. For C_27_H_26_N_4_O_4_S (502.58): C, 64.52; H, 5.21; N, 11.15; S, 6.38; Found: C, 64.37; H, 5.44; N, 11.00; S, 6.49.

*Methyl-2-[2-(5-benzyl-4-phenyl-4H-[1,2,4]triazol-3-thio)-acetylamino]-3-(1H-indol-3-yl)-propionate* (**6h**)**.** From l-TrpOCH_3_·HCl (**5h**, 0.20 g). Colorless crystals (0.36 g, 69%); mp 111 °C, IR (KBr disk): 3391, 3265, 3063, 2958, 1742, 1690, 1678, 1647, 1611, 1552, 1507 cm^-1^; ^1^H-NMR (200 MHz, CDCl_3_ *δ* ppm): 8.70 (1H, bs, D_2_O exchangeable, NH); 8.31 (1H, bs, D_2_O exchangeable, NH); 7.85-7.73 (4H, m, ArH); 7.61-7.53 (6H, m, ArH); 7.46-7.24 (5H, m, ArH); 5.24-5.18 (1H, m, NHCH); 4.33 (2H, s, SCH_2_); 4.16 (2H, s, PhCH_2_); 4.00 (3H, s, OMe); 3.68-3.64 (2H, d, *J* = 6.8 Hz, CH_2_CH). Anal. Calcd. For C_29_H_27_N_5_O_3_S (525.62): C, 66.27; H, 5.18; N, 13.32; S, 6.10; Found: C, 66.01; H, 4.99; N, 13.53; S, 5.90. 

### 3.5. *General procedure for preparation of hydrazides **7a,b***

To a solution of esters **6a,b** (1.0 mmol) in ethyl alcohol (30 mL), hydrazine hydrate (0.24 mL, 5.0 mmol) were added. The reaction mixture was refluxed for 4h, cooled; the precipitated white precipitate was filtered and crystallized from aq. EtOH.

*N-2-Hydrazinocarbonyl-ethyl-2-(5-benzyl-4-phenyl-4H-[1,2,4]triazol-3-thio)-acetamide* (**7a**). From ester **6a** (0.41 g). Colorless crystals (0.37 g, 90 %); 139–140 °C, IR (KBr disk): 3317, 3295, 3223, 3209, 3065, 2957, 1683, 1671, 1660, 1648, 1601, 1545, 1513 cm^-1^; ^1^H-NMR (200 MHz, CDCl_3_ *δ* ppm): 8.16 (1H, bs, D_2_O exchangeable, NH); 7.51-7.33( 4H, m, ArH); 7.27-7.15 (4H, m, ArH); 7.10 (2H, d, *J* = 8.2 Hz, ArH); 6.58 (1H, bs, D_2_O exchangeable, NH); 4.49 (2H, m, NHCH_2_); 4.29 (2H, bs, D_2_O exchangeable, NH_2_); 3.99 (2H, s, SCH_2_); 3.86 (2H, s, PhCH_2_); 2.39 (2H, t, *J* = 6.0 Hz, NCH_2_CH_2_). Anal. Calcd. For C_20_H_22_N_6_O_2_S (410.49): C, 58.52; H, 5.40; N, 20.47; S, 7.81; Found: C, 58.84; H, 5.18; N, 20.11; S, 7.66.

*N-Hydrazinocarbonylmethyl-2-(5-benzyl-4-phenyl-4H-[1,2,4]triazol-3-thio)-acetamide* (**7b**). From ester **6b** (0.40 g). Colorless crystals (0.37 g, 93%); mp 152–153 °C, IR (KBr disk): 3302, 3291, 3218, 3202, 3071, 2962, 1688, 1669, 1671, 1641, 1600 cm^-1^; ^1^H-NMR (200 MHz, CDCl_3_ *δ* ppm): 8.45 (1H, bs, D_2_O exchangeable, NH); 7.53-7.49 (4H, m, ArH); 7.33-7.27 (4H, m, ArH); 7.17 (2H, d, *J* = 8.2 Hz, ArH); 6.64 (1H, bs, D_2_O exchangeable, NH); 4.60 (2H, d, *J* = 7.4 Hz, NHCH_2_); 4.30 (2H, bs, D_2_O exchangeable, NH_2_); 4.11 (2H, s, SCH_2_); 3.98 (2H, s, PhCH_2_). Anal. Calcd. For C_19_H_20_N_6_O_2_S (396.47): C, 57.56; H, 5.08; N, 21.20; S, 8.09; Found: C, 57.33; H, 5.02; N, 21.47; S, 8.44. 

### 3.6. *General procedure for preparation of **10a-e***

Dipeptides **10a-e** were prepared according to the previously described azide procedure.

*Methyl-2-(2-[2-(5-benzyl-4-phenyl-4H-[1,2,4]triazol-3-thio)-acetylamino]-acetylamino)-acetate* (**10a**). From hydrazide **7b** (0.40 g) and GlyOCH_3_·HCl (**9a**, 0.13 g). Colorless crystals (0.22 g, 49 %); mp 78–80 °C, IR (KBr disk): 3207, 3198, 3067, 2975, 1701, 1686, 1674, 1665, 1614 cm^-1^; ^1^H-NMR (200 MHz, CDCl_3_ *δ* ppm): 8.47 (1H, bs, D_2_O exchangeable, NH); 7.55-7.48 (4H, m, ArH); 7.30-7.24 (4H, m, ArH); 7.19 (2H, d, *J* = 8.2 Hz, ArH); 6.63 (1H, bs, D_2_O exchangeable, NH); 4.57 (2H, d, *J* = 7.4 Hz, NHCH_2_); 4.52 (2H, d, *J* = 7.4 Hz, NHCH_2_); 4.06 (2H, s, SCH_2_); 3.86 (2H, s, PhCH_2_); 4.76 (3H, s, OMe). ^13^C-NMR (CDCl_3_, 75 MHz, *δ* ppm) 32.1, 37.2, 45.4, 47.6, 52.4, 128.1, 128.2, 128.5, 128.8, 129.1, 129.8, 130.3, 135.1, 143.9, 157.6, 168.7, 170.0, 172.9; Anal. Calcd. For C_22_H_23_N_5_O_4_S (453.51): C, 58.26; H, 5.11; N, 15.44; S, 7.07; Found: C, 58.02; H, 5.45; N, 15.38; S, 6.82.

*Methyl-3-(2-[2-(5-benzyl-4-phenyl-4H-[1,2,4]triazol-3-thio)-acetylamino]-acetylamino)-propionate* (**10b**). From hydrazide **7b** (0.40 g) and β−AlaOCH_3_·HCl (**9b**, 0.14 g). Colorless crystals (0.26 g, 56 %); mp 73–74 °C, IR (KBr disk): 3179, 3167, 3061, 2968, 1703, 1690, 1676, 1667, 1610 cm^-1^; ^1^H-NMR (200 MHz, CDCl_3_ *δ* ppm): 8.52 (1H, bs, D_2_O exchangeable, NH); 7.49-7.45 (4H, m, ArH); 7.29-7.23 (4H, m, ArH); 7.16 (2H, d, *J* = 8.0 Hz, ArH); 6.61 (1H, bs, D_2_O exchangeable, NH); 4.63 (2H, d, *J* = 7.4 Hz, NHCH_2_); 4.06 (2H, s, SCH_2_); 3.86 (2H, s, PhCH_2_); 3.73 (3H, s, OMe); 3.54-3.49 (2H, m, NHCH_2_); 2.91 (2H, t, *J* = 7.2 Hz, NCH_2_CH_2_). ^13^C-NMR (CDCl_3_, 75 MHz, *δ* ppm): *δ* 27.5, 33.3, 38.2, 39.6, 45.8, 51.4, 127.3, 128.0, 128.3 128.7, 129.3, 130.7, 132.2, 139.0, 145.6, 160.2, 168.8, 172.6, 173.1; Anal. Calcd. For C_23_H_25_N_5_O_4_S (467.54): C, 59.08; H, 5.39; N, 14.98; S, 6.86; Found: C, 58.87; H, 5.18; N, 14.72; S, 6.54. Mass spectrum, m/z (%): 468(2), 467(11), 381(22), 352(27), 281(19), 280(34), 266(26), 267(61), 234(32), 235(41), 91(100).

*Methyl-2-(3-[2-(5-benzyl-4-phenyl-4H-[1,2,4]triazol-3-thio)-acetylamino]-propionylamino)-acetate* (**10c**). From hydrazide **7a** (0.41 g) and GlyOCH_3_·HCl (**9a**, 0.13 g). colorless crystals (0.21 g, 45 %); 77–78 °C, IR (KBr disk): 3183, 3177, 3067, 2956, 1699, 1687, 1672, 1651, 1610 cm^-1^; ^1^H-NMR (200 MHz, CDCl_3_ *δ* ppm): 8.22 (1H, bs, D_2_O exchangeable, NH); 7.57-7.36 (4H, m, ArH); 7.29-7.19 (4H, m, ArH); 7.14 (2H, d, *J* = 8.2 Hz, ArH); 6.51 (1H, bs, D_2_O exchangeable, NH); 4.62 (2H, m, NHCH_2_); 4.08 (2H, s, SCH_2_); 3.97 (2H, s, PhCH_2_); 3.73 (3H, s, OMe); 3.57 (2H, m, NHCH_2_); 2.39 (2H, t, *J* = 7.0 Hz, NCH_2_CH_2_). ^13^C-NMR (CDCl_3_, 75 MHz, *δ* ppm): *δ* 27.8, 33.9, 36.3, 38.0, 39.5, 45.1, 51.0, 127.1, 128.2, 128.7 128.9, 129.7, 130.3, 132.0, 139.3, 145.1, 159.4, 170.1, 171.9, 172.8; Anal. Calcd. For C_23_H_25_N_5_O_4_S (467.54): C, 59.08; H, 5.39; N, 14.98; S, 6.86; Found: C, 59.31; H, 5.52; N, 14.67; S, 6.90.

*Methyl-3-(3-[2-(5-benzyl-4-phenyl-4H-[1,2,4]triazol-3-thio)-acetylamino]-propionylamino)-propionate* (**10d**)**.** From hydrazide **7a** (0.41 g) and β−AlaOCH_3_·HCl (**9b**, 0.14 g). Colorless crystals (0.20 g, 37 %); 71–73 °C, IR (KBr disk): 3191, 3180, 3062, 2955, 1704, 1689, 1666, 1647, 1607 cm^-1^; ^1^H-NMR (200 MHz, CDCl_3_ *δ* ppm): 8.29 (1H, bs, D_2_O exchangeable, NH); 7.61-7.52 (4H, m, ArH); 7.24-7.08 (4H, m, ArH); 7.01 (2H, d, *J* = 8.2 Hz, ArH); 6.44 (1H, bs, D_2_O exchangeable, NH); 4.08 (2H, s, SCH_2_); 3.97 (2H, s, PhCH_2_), 3.73 (3H, s, OMe); 3.54 (2H, m, NHCH_2_); 3.52-3.47 (2H, m, NHCH_2_); 2.39-2.32 (4H, m, 2 CH_2_). Anal. Calcd. For C_24_H_27_N_5_O_4_S (481.57): C, 59.86; H, 5.65; N, 14.54; S, 6.66; Found: C, 59.58; H, 5.92; N, 14.20; S, 6.97.

*Methyl-2-(3-[2-(5-benzyl-4-phenyl-4H-[1,2,4]triazol-3-thio)-acetylamino]-propionylamino)-3-hydroxy-propionate* (**10e**). From hydrazide **7a** (0.41 g) and l-SerOCH_3_·HCl (**9e**, 0.16 g). Colorless crystals (0.24g, 48 %); 79–81 °C, IR (KBr disk): 3374, 3207, 3191, 3047, 2976, 1707, 1691, 1683, 1664, 1601 cm^-1^; ^1^H-NMR (200 MHz, CDCl_3_ *δ* ppm): 8.32 (1H, bs, D_2_O exchangeable, NH); 7.54-7.46 (4H, m, ArH); 7.31-7.27 (4H, m, ArH); 7.13 (2H, d, *J* = 8.2 Hz, ArH); 6.37 (1H, bs, D_2_O exchangeable, NH); 5.04-5.00 (1H, m, NHCH); 4.29-4.26 (2H, m, NHCH_2_); 4.02 (2H, s, SCH_2_); 3.95 (2H, s, PhCH_2_); 3.77 (3H, s, OMe); 3.53-3.50 (2H, m, CHCH_2_OH); 3.32 (1H, bs, D_2_O exchangeable, CH_2_OH); 2.35-2.32 (2H, m, NCH_2_CH_2_). Anal. Calcd. For C_24_H_27_N_5_O_5_S (497.57): C, 57.93; H, 5.47; N, 14.08; S, 6.44; Found: C, 57.61; H, 5.42; N, 14.27; S, 6.28.

### 3.7. General procedure for azide Curtius rearrangement to the corresponding isocyanate; preparation of ***11-14***

To a cold solution (−5 °C) of hydrazide **3** (0.34 g, 1.0 mmol) in acetic acid (6 mL), 1 N HCl (3 mL), and water (25 mL) was added a solution of NaNO_2_ (0.87 g, 1.0 mmol) in cold water (3 mL). The reaction mixture was stirred at −5 °C for 15 min. The yellow syrup formed was extracted with cold benzene (30 mL), washed with cold 3% NaHCO_3_, H_2_O and finally dried (Na_2_SO_4_); the extract was filtered off and refluxed for 2 h. To this solution the appropriate amount of amine and/or MeOH (1.0 mmol) in benzene (20 mL) was added. The reflux was continued for an additional 2 h. The solvent was evaporated under reduced pressure and the residue was triturated with petroleum ether and crystallized from petroleum ether/ethyl acetate to give the desired product.

*3-Cyclohexyl-1-(5-benzyl-4-phenyl-4H-[1,2,4]triazol-3-thiomethyl)-urea* (**11**). From cyclohexylamine (0.10 g). Colorless crystals (0.35 g, 83%); mp 99 °C, IR (KBr disk): 3218, 3201, 3091, 2910, 1710, 1676, 1643, 1603, 1571, 1509 cm^-1^; ^1^H-NMR (200 MHz, CDCl_3_ *δ* ppm): 10.48 (1H, bs, D_2_O exchangeable, NH); 7.70-7.61 (4H, m, ArH); 7.47-7.32 (2H, d, *J* = 8.4 Hz, ArH); 7.21-6.98 (4H, m, ArH); 6.90 (1H, bs, D_2_O exchangeable, NH); 3.87 (2H, s, SCH_2_); 3.80 (2H, s, PhCH_2_); 2.40 (1H, m, NHCH); 1.73-1.62 (4H, m, 2 CH_2_); 1.33-1.27 (2H, m, (CH_2_)_2_CH_2_); 0.96-0.84 (4H, m, 2 CH_2_) Anal. Calcd. For C_23_H_27_N_5_OS (421.56): C, 65.53; H, 6.46; N, 16.61; S, 7.61; Found: C, 65.31; H, 6.71; N, 16.49; S, 7.81. Mass spectrum, m/z (%): 422(4), 421(13), 393(18), 344(21), 330(31), 322(21), 281(16), 280(37), 266(24), 267(55), 234(29), 235(44), 91(100).

*1-(5-Benzyl-4-phenyl-4H-[1,2,4]triazol-3-thiomethyl)-3-(4-chlorophenyl)-urea* (**12a**)**.** From *p*-chloro-aniline (0.13 g). Colorless crystals (0.34 g, 76%); mp 113 °C, IR (KBr disk): 3212, 3145, 3042, 2960, 1721, 1682, 1644, 1606, 1543, 1503 cm^-1^; ^1^H-NMR (200 MHz, CDCl_3_ *δ* ppm): 10.29 (1H, bs, D_2_O exchangeable, NH); 7.41-7.31 (4H, m, ArH); 7.18-7.09 (2H, d, *J* = 8.4 Hz, ArH); 7.10-6.77 (8H, m, ArH); 5.64 (1H, bs, D_2_O exchangeable, NH); 4.03 (2H, s, SCH_2_); 3.80 (2H, s, PhCH_2_). Anal. Calcd. For C_23_H_20_ClN_5_OS (449.96): C, 61.39; H, 4.48; N, 15.56; S, 7.13; Found: C, 61.11; H, 4.62; N, 15.41; S, 7.39.

*1-(5-Benzyl-4-phenyl-4H-[1,2,4]triazol-3-thiomethyl)-3-(4-nitrophenyl)-urea* (**12b**). From *p*-nitro-aniline (0.14 g). Yellow crystals (0.31 g, 67%); mp 127 °C, IR (KBr disk): 3233, 3164, 3049, 2953, 1715, 1673, 1652, 1613, 1552, 1523, 1500 cm^-1^; ^1^H-NMR (200 MHz, CDCl_3_ *δ* ppm): 10.11 (1H, bs, D_2_O exchangeable, NH); 7.66 (2H, d, 8.0, ArH); 7.46-7.41 (4H, m, ArH); 7.31-7.27 (2H, d, *J* = 8.0 Hz, ArH); 7.11-6.87 (6H, m, ArH); 5.64 (1H, bs, D_2_O exchangeable, NH); 4.21 (2H, s, SCH_2_); 3.83 (2H, s, PhCH_2_). Anal. Calcd. For C_23_H_20_N_6_O_3_S (460.51): C, 59.99; H, 4.38; N, 18.25; S, 6.96; Found: C, 59.81; H, 4.02; N, 18.47; S, 6.89.

*Morpholine-4-carboxylic acid (5-benzyl-4-phenyl-4H-[1,2,4]triazol-3-thiomethyl)-amide* **(13a**). From morpholine (0.09 g). Colorless crystals (0.30 g, 73%); mp 91 °C, IR (KBr disk): 3242, 3081, 2968, 1706, 1678, 1646, 1605, 1556, 1511 cm^-1^; ^1^H-NMR (200 MHz, CDCl_3_ *δ* ppm): 10.36 (1H, bs, D_2_O exchangeable, NH); 7.47-7.31 (4H, m, ArH); 7.20-7.01 (2H, d, *J* = 8.4 Hz, ArH); 6.97-6.87 (4H, m, ArH), 4.06 (2H, s, SCH_2_); 3.84 (2H, s, PhCH_2_); 3.72-3.66 (4H, m, O(CH_2_)_2_); 3.5-3.43 (4H, m, NH(CH_2_)_2_), ^13^C-NMR (CDCl_3_, 75 MHz, *δ* ppm) 34.5, 46.8, 47.4, 68.1, 127.4, 127.7, 127.9, 128.5, 128.7, 128.8, 129.1, 136.1, 146.2, 155.7, 159.4; Anal. Calcd. For C_21_H_23_N_5_O_2_S (409.50): C, 61.59; H, 5.66; N, 17.10; S, 7.83; Found: C, 61.42; H, 5.49; N, 17.28; S, 7.97.

*Piperidine-1-carboxylic acid (5-benzyl-4-phenyl-4H-[1,2,4]triazol-3-thiomethyl)-amide* (**13b**). From piperdine (0.09 g). Colorless crystals (0.35 g, 83%); mp 109 °C, IR (KBr disk): 3214, 3174, 2959, 1705, 1681, 1640, 1624, 1545, 1501 cm^-1^; ^1^H-NMR (200 MHz, CDCl_3_ *δ* ppm): 10.59 (1H, bs, D_2_O exchangeable, NH); 7.45-7.41 (4H, m, ArH); 7.19 (2H, d, *J* = 8.4 Hz, ArH); 7.09-6.90 (4H, m, ArH); 4.04 (2H, s, SCH_2_); 3.80 (2H, s, PhCH_2_); 3.6-3.53 (4H, m, N(CH_2_)_2_); 2.07 (2H, m, (CH_2_)_2_(CH_2_)); 1.61-1.43 (4H, m, 2 CH_2|_). Anal. Calcd. For C_22_H_25_N_5_OS (407.53): C, 64.84; H, 6.18; N, 17.18; S, 7.87; Found: C, 64.99; H, 6.31; N, 17.01; S, 7.63. Mass spectrum, m/z (%): 408(3), 407(11), 379(29), 322(34), 281(21), 280(32), 266(17), 267(58), 234(27), 235(36), 91(100).

*(5-Benzyl-4-phenyl-4H-[1,2,4]triazol-3-thiomethyl)-carbamic acid methyl ester* (**14**). From MeOH (0.04 g). Colourless crystals (0.22 g, 62%); mp 54 °C, IR (KBr disk): 3178, 3065, 2942, 1713, 1667, 1639, 1604, 1531, 1500 cm^-1^; ^1^H-NMR (200 MHz, CDCl_3_ *δ* ppm): 10.05 (1H, bs, D_2_O exchangeable, NH); 7.44-7.39 (4H, m, ArH); 7.30-7.26 (2H, d, *J* = 8.0 Hz, ArH); 7.11-6.86 (4H, m, ArH); 4.05 (2H, s, SCH_2_); 3.87 (2H, s, PhCH_2_); 3.77 (3H, s, OMe). ^13^C-NMR (CDCl_3_, 75 MHz, *δ* ppm): *δ* 29.1, 48.4, 50.2, 127.6, 128.4, 128.8, 129.1, 129.6, 131.0, 132.6, 139.2, 146.0, 159.9, 160.4; Anal. Calcd. For C_18_H_18_N_4_O_2_S (354.43): C, 61.00; H, 5.12; N, 15.81; S, 9.05; Found: C, 59.68; H, 5.18; N, 15.69; S, 9.37.

## 4. Conclusions

In summary; twenty three newly triazoles derivatives were synthesized and their chemical structure were elucidated via different analytical and spectroscopic methods. The antimicrobial activities of some of these compounds against two bacterial colonies (*Escherechia coli* and *Bacillus subtilis*) and two fungal cultures (*Phytophthora infestans* and *Colletotricum gloeosporioides*) were studied. The synthesized compounds **12b, 6c, 2** and **14** showed important biological activity. 

## Figures and Tables

**Table 1:** Antimicrobial activity of triazole derivatives.

Comp. No.	Antifungal		Antibacterial	
	*Phytophthora infestans*	*Colletotricum gloeosporioides*	*Bacillus subtilis*	*Escherichia coli*
**2**	++	-	-	-
**3**	-	-	-	-
**6a**	-	-	-	-
**6b**	-	-	-	-
**6c**	-	-	+++	-
**6d**	-	-	-	-
**6e**	-	-	-	-
**6g**	-	-	-	-
**6h**	-	-	-	-
**6f**	-	-	-	-
**11**	-	-	-	-
**12a**	-	-	-	-
**12b**	-	++	+	-
**13a**	-	-	-	-
**13b**	-	-	-	-
**14**	-	-	-	+
